# Ferulic Acid: A Hope for Alzheimer’s Disease Therapy from Plants

**DOI:** 10.3390/nu7075246

**Published:** 2015-07-15

**Authors:** Antonella Sgarbossa, Daniela Giacomazza, Marta di Carlo

**Affiliations:** 1 NEST Istituto Nanoscienze CNR and Scuola Normale Superiore, Piazza S. Silvestro 12, Pisa 56127, Italy; E-Mail: antonella.sgarbossa@nano.cnr.it; 2 Istituto di Biofisica (IBF) CNR, Via U. La Malfa 153, Palermo 90146, Italy; E-Mail: daniela.giacomazza@cnr.it; 3 Istituto di Biomedicina ed Immunologia Molecolare (IBIM) CNR, Via U. La Malfa 153, Palermo 90146, Italy

**Keywords:** antioxidants, Alzheimer’s disease, fibrillogenesis, oxidative stress, apoptosis, nanotechnology

## Abstract

Alzheimer’s disease (AD) is a neurodegenerative disorder characterized by the deposition of extracellular amyloid-beta peptide (Aβ) and intracellular neurofibrillar tangles, associated with loss of neurons in the brain and consequent learning and memory deficits. Aβ is the major component of the senile plaques and is believed to play a central role in the development and progress of AD both in oligomer and fibril forms. Inhibition of the formation of Aβ fibrils as well as the destabilization of preformed Aβ in the Central Nervous System (CNS) would be an attractive therapeutic target for the treatment of AD. Moreover, a large number of studies indicate that oxidative stress and mitochondrial dysfunction may play an important role in AD and their suppression or reduction via antioxidant use could be a promising preventive or therapeutic intervention for AD patients. Many antioxidant compounds have been demonstrated to protect the brain from Aβ neurotoxicity. Ferulic acid (FA) is an antioxidant naturally present in plant cell walls with anti-inflammatory activities and it is able to act as a free radical scavenger. Here we present the role of FA as inhibitor or disaggregating agent of amyloid structures as well as its effects on biological models.

## 1. Introduction

Ferulic acid (4-hydroxy-3-methoxycinnamic acid, FA) is a widely distributed constituent of plants. It was first isolated from *Ferula foetida*, the plant from which its name has been derived, in 1866. It is most abundant in cereal grains where FA can reach the concentration of 2 g/kg dry weight [1,2]. The metabolism of FA in plants starts with the aromatic amino acids through the shikimate pathway. The production of *p*-coumaric acid, from phenylalanine or tyrosine, leads to the formation of caffeic acid, which, in turn, is converted into FA after methylation reaction [3]. Together with pentosan chains, arabinoxylans, and hemicelluloses, FA becomes part of the lignin polymer [4], thus conferring rigidity to cell walls. It can be found free, in dimerized form, or esterified with proteins and polysaccharides in a wide variety of natural products [5].

FA exhibits several physiological functions in plants: protection of cells against hydrolytic enzymes during germination [6]; regulation of plant growth [7]; inhibition of competing plants [7]; and uptake of minerals and water in roots [8]. Furthermore, it has been shown that FA protects cereals against aphids [9], insects [10], and fungal infections [11].

The daily intake of caffeic acid and FA has been estimated to be about 500–1000 mg in humans consuming fruits, vegetables, cereal bran, beer, and coffee [12]. Free phenolic acids can be absorbed by the monocarboxylic acid transporters (MCT) in the gastrointestinal mucosa. The absorption efficiency greatly depends on the affinity of the phenolic compounds for MCT and the administration of different phenolic acids in rats has revealed that the affinity scale is gallic acid = chlorogenic acid < caffeicacid < *p*-coumaric acid = FA [13]. The plasma peak for FA was achieved in 5–10 min after oral administration [13,14].

*In vivo* experiments on rats have shown that the metabolism of esterified FA occurs first with a de-esterification reaction done by the enzymes produced by the lactic acid bacteria present in the gastrointestinal tract [15]. Then, FA is converted into a variety of metabolites, predominantly containing glucuronic or sulphate molecules [16,17,18]. Jacobson *et al.* [19] identified FA, vanillic acid, and caffeic acid in human urine after ingestion of 1 g of FA. It has also been evidenced that free FA or FA linked to simple sugars had a higher absorption rate when compared to FA bound with more complex matrices: in humans, the urinary recovery of FA was 74% after drinking beer while it was in the range of 11%–25% after tomato consumption [20,21]. This significant difference is due to the fact that in the beer FA is found as free, while in tomato it is present as FA-*O*-glucoside [22].

The conjugation of FA mainly occurs in liver through the enzymes sulfotransferases and a Uridine 5′-diphospho-glucuronosyltransferase (UDP-glucuronosyltransferases) [23], although the kidney [17] and the intestinal mucosa [24] can participate in the process.

The purification of FA from natural sources is not easy because FA is covalently linked to lignin and other biopolymers. Recently, an enzymatic production of FA from defatted rice bran has been developed by using a combination of bacterial enzymes [25].

From a chemical point of view, FA belongs to the broad class of phenols, in particular to the hydroxycinnamic acids. They can exist both in cis and trans forms, the latter being the most stable and diffused in nature [26]. After Ultra Violet irradiation, each form can be converted into the other one [26].

**Figure 1:**
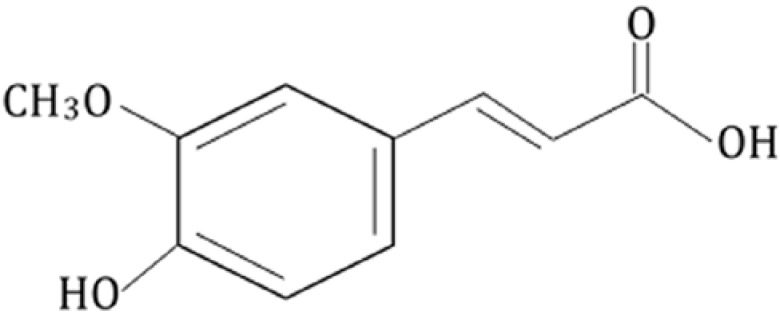
Chemical structure of ferulic acid.

Due to its chemical structure (Figure 1), FA possesses several structural motifs that confer on it a strong free radical scavenging character [27]: (i) it is capable of forming a stabilized resonance phenoxy radical since the unpaired electron is delocalized across the whole molecule; (ii) the collision with a ferulate radical leads to the production of a curcuma molecule, thus increasing the scavenging activity of the compound due to the occurrence of additional resonance stabilization; (iii) the tertiary structure obtained by the carboxylic acid group with adjacent unsaturated C-C double bond can stabilize free radicals via resonance or by offering additional sites to prevent free radical membrane attack, and furthermore; (iv) this carboxylic acid group can act as a lipid anchor, thereby affording protection against lipid peroxidation [28].

**Figure 2:**
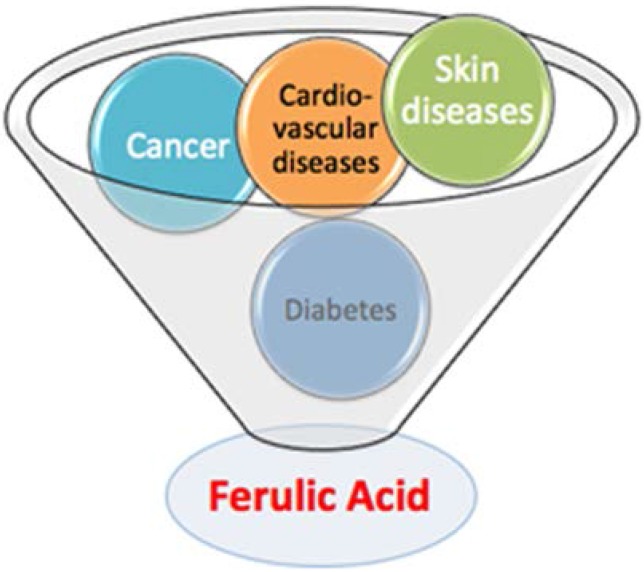
Some pathologies against which ferulic acid has given promising results.

The antioxidant effect of FA has been verified against several acute and chronic pathologies such as intestinal ischemia [29], cancer [30,31], cardiovascular [32] and skin diseases [33], diabetes [34] (Figure 2), cochlear oxidative damage due to repeated noise exposure [35], and oxidative cellular stress in human dermal fibroblasts [36]. In addition, the free radical scavenging activity of FA has been tested against several neurodegenerative pathologies, Alzheimer’s disease (AD) in particular.

AD is the main cause of the dementia syndrome whose incidence and frequency increase with age. Since the elderly population is growing worldwide, AD is quickly becoming one of the major universal healthcare problems [37]. Currently, however, there are neither precise diagnostic approaches nor effective therapeutic agents available for AD. The degeneration of nervous tissue begins many years or even decades before the patient experiences any of the AD symptoms and the currently available therapeutics for AD can simply act in decreasing its symptoms. Drugs belonging to the families of acetylcholinesterase inhibitors (donepezil, rivastigmine) or *N*-methyl-d-aspartate (NMDA) receptor, glutamate receptor antagonists (galantamine and memantine), have been administered to attenuate AD symptoms and have shown positive effects against the disease along with heavy side effects [38]. Other drugs are under development at the moment [38].

In the current review we will focus on the effects of FA, illustrating the role of FA as inhibitor or disaggregating agent of amyloid structures as well as its effects on biological models.

## 2. Biophysical Study on Ferulic Acid

### 2.1 Amyloid Fibrillogenesis

The overproduction and aberrant self-assembly of the amyloid β peptide (Aβ), formed by the proteolitic cleavage of the larger Amyloid Protein Precursor (APP) by β- and γ-secretases, into fibrillar aggregates constitutes the first steps of the so-called amyloid cascade hypothesis, thought to trigger AD. Fibril formation is accompanied by the production of metastable oligomeric species considered as the primary pathogenic agents. These extremely toxic oligomers have high hydrophobicity, are small [39], and constitute a heterogeneous group characterized by several highly dynamic different assemblies with multiple conformational states. Although the mechanism of cytotoxicity is not yet fully understood, it has been ascertained that amyloid oligomers are the most toxic species [40]; in fact, they directly interact with and affect cell plasma membranes by forming pores and consequently disrupting several cellular processes. Amyloid fibrils have also been recently demonstrated to modify the membrane integrity. In fact, they interact with lipid bilayers and are destabilized and disassembled in the pre-fibrillar toxic forms, inducing cell dysfunction (although to a lesser extent) [41,42,43,44].

**Figure 3:**
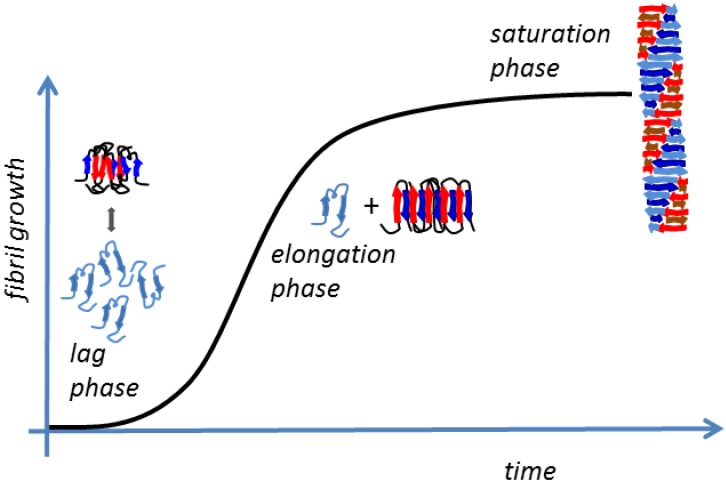
Schematic representation of the amyloid fibrillation.

The self-assembly of Aβ peptides in well-ordered fibrils constituting the senile plaques found in AD brains is a complex process composed by several steps. It is characterized by multiple transitional aggregation species as initial seeds, soluble small oligomers, protofibrils, and insoluble amyloid fibrils, with a β-sheet conformation. The kinetics of amyloid formation are best described by a sigmoid curve and can be schematically subdivided in three stages [45] as follows (Figure 3):

(1) The slow lag nucleation phase, in which monomers gradually undergo a secondary structure conformational change from random coil to β-sheet and associate to form oligomeric nuclei/protofibrils;
(2) The fast exponential elongation phase, in which the soluble species are progressively arranged at the ends of preformed β-sheet-rich structures in a thermodynamically favorable process. The initial oligomeric nuclei rapidly grow by further addition of monomers forming larger fibrils;
(3) The saturation phase, in which the fibrils are completely formed and associate with each other, giving rise to stable mature fibers.

Amyloid fibril formation is a first-order phase transition limited by the rate of the monomer formation. Its kinetic curve can be fitted by the Finke-Watzky mechanism of slow continuous nucleation, A→B (rate constant k_1_), followed by typically fast, autocatalytic surface growth, A + B→2B (rate constant k_2_) [46].

The driving intermolecular forces that promote and favor Aβ self-assembly are common to all proteins, without any meaningful dependence on the specific peptide sequence (hydrophobic interactions, backbone hydrogen bonding, stacking interactions).

There is a remarkable occurrence of aromatic residues in unrelated amyloid-forming proteins. Furthermore, short fragments of Aβ containing two phenylalanine residues (QKLVFF) were found to self-assemble and also to bind specifically to full-length peptides. These observations supported the hypothesis that π-π interactions may play a central role in the molecular recognition and Aβ self-assembly process. Intermolecular stacking interactions between aromatic residues may accelerate the self-assembly process, providing directionality and an energetic contribution to the aggregation process [47].

Aromatic interactions are weak (of the order of a few kcal·mol^−1^) non-covalent interactions between two molecules consisting of a number of components, including electrostatic interactions (between charges, dipoles, quadrupoles, and higher multipoles of both subsystems, proportional to the product of the multipoles and a first or higher power of reciprocal distance), van der Waals forces (resulting from the sum of the dispersion and repulsion energies), and solvophobic interactions. The electrostatic component of these non-covalent interactions accounts for mutual orientation patterns in aromatic-aromatic interactions, including the edge-to-face, commonly observed between aromatic residues in proteins, and the offset stacked geometries, which is also normally observed in proteins and forms the geometry of base stacking in DNA [48].

### 2.2 Small Natural Molecule Inhibitors

As already mentioned, the concept of aromatic interactions as a driving force for amyloid formation plays an important role in the development and the study of small phenolic inhibitors. This approach was initially based on early findings showing that small aromatic molecules such as Congo red and Thioflavin T (ThT) interact specifically with amyloid fibril [49,50]. In the past few years, a wide variety of small aromatic molecules, natural or synthetic, have been reported as inhibitors of amyloid fibril formation. Natural aromatic molecules, which represent the major phytochemical components of fruit, vegetables, beverages, and grains, have recently received increasing attention for their anti-aging effects and benefits for human health mainly due to significant free radical scavenging properties and antioxidant activity. They are characterized by the presence of one or more phenolic rings able to interact with the aromatic residues of the amyloid peptides, undermining the well-ordered and compact packing of the self-assembly process. The formation of small molecule-amyloid peptide complexes may also be favored by weak, non-covalent forces such as electrostatic, van der Waals, hydrophobic, and solvophobic forces [51,52,53].

In order to explain phenolic activity, several mechanisms have been proposed to date and structural similarities between various highly efficient inhibitors have been identified. It has become apparent that the presence of phenolic rings with a few linkers and at least two Hydroxyl groups (OH) could favor effective non-covalent interactions with the fibril β-sheet structures and interfere with their elongation and/or assembly [54,55,56,57]. According to Gazit *et al.* [52], ligands with specific structural conformations and definite orientations of their phenolic moieties, in relation to the aromatic residues of the amyloidogenic sequence, have the tendency to destabilize the oligopeptide complexes and hinder further peptide aggregation.

On the basis of the experimental data available, the anti-amyloidogenic behavior of phenols could be accomplished in three possible ways: (1) inhibition of the initial stage of the self-assembly process leading to oligomeric species; (2) inhibition of the elongation of protofibrils and extension in fibrils; (3) destabilization, disaggregation, and/or fragmentation of the mature fibrils and their conversion to amorphous structures.

Among these compounds, FA (4-hydroxy-3-methoxycinnamic acid) represents one of the most abundant phenols. As highlighted before, its potent antioxidant and anti-inflammatory properties make FA an interesting and promising candidate for prevention and/or treatment of disorders linked to oxidative stress, including AD [58,59].

### 2.3. Ferulic Acid and Amyloid Aggregation

FA contains one phenolic ring and is one of the metabolites of the curcuma, which has been demonstrated to possess neuroprotective capabilities resulting from its ability to directly alter the kinetics of Aβ fibril formation, as well as its anti-oxidative and anti-inflammatory properties [60]. On account of FA’s structural similarity to curcuma, it was postulated that FA could be a suitable molecule for specifically binding to Aβ and inhibiting fibril formation. Furthermore, this compact structure could be also suitable for specific interactions with Aβ mature fibrils, possibly promoting their consequent destabilization.

By means of *in vitro* fluorescence spectroscopic studies with Thioflavin T and electron microscopic analysis, the effects of FA on the formation, extension, and destabilization of Aβ_(1–40)_ and Aβ_(1–42)_ fibrils were examined. It was shown that FA, dose-dependently, inhibited fibril formation as well as extension and destabilized preformed fibrils. The anti-amyloidogenic and fibril-destabilizing activities of FA were reported to be slightly weaker than the inhibitory effects of curcuma. On the basis of these results it was speculated that “FA could prevent the development of AD, not only through scavenging reactive oxygen species, but also through direct inhibition of the deposition of fibrils in the brain” [61].

In a subsequent study aimed at systematically investigating the inhibitory effects of phenolic compounds on Aβ aggregation *in vivo*, several molecules, including FA, were fed to AD model transgenic mice (Tg2576) and the cerebral plaque burden as well as the formation of Aβ oligomers was compared [62]. In previous studies, long-term administration of FA was reported to protect mice against Aβ-induced learning and memory deficits *in vivo* [63,64] and FA was shown to protect neurons against Aβ-induced oxidative stress and neurotoxicity *in vitro* [65], but the oral administration of FA did not show any significant effect on Aβ oligomers or Aβ deposition *in vivo* [60,62].

These unexpected results have been recently revised by the same authors. They studied Aβ_(1–42)_ and Aβ_(1–40)_ self-assembly in the presence of five phenolic compounds, myricetin (Myr), FA, nordihydroguaiaretic acid (NDGA), curcuma (Cur), and rosmarinic acid (RA) using several well-established techniques for studying amyloid formation: photo-induced cross-linking of unmodified proteins (PICUP), atomic force microscopy (AFM), circular dichroism (CD) spectroscopy, and nuclear magnetic resonance (NMR). Furthermore, the effects on Aβ assembly-induced cytotoxicity and synaptic dysfunction were examined by means of 3-(19)-2,5-diphenyltetrazolium bromide (MTT) assays and electrophysiological assays for long-term potentiation (LTP) and depression (LTD) in hippocampal slices derived from C57BL/6 mice. It was demonstrated that all phenolic compounds, including FA, prevented low-order (10 monomers) oligomer formation. The authors attributed the discrepancy between these results and their previous results to the nonspecific nature of the A11 antibody that was used to detect oligomers in the Tg2576 mice, particularly when compared with cross-linked oligomers detected by PICUP. In addition, the MTT, LTP, and LTD assays established that the phenols inhibit Aβ oligomer-induced cellular and synaptic toxicities [66].

In the same year, other authors conducted *in vivo* studies in a transgenic nematode *Caenorhabditis elegans* model that expresses the human Aβ_(1–42)_ within the body wall muscles, resulting in their paralysis. FA was found to decrease the amount of oligomeric species *in vitro* and to protect *Caenorhabditis elegans* against Aβ toxicity, prolonging nematode survival more than morin, quercetin, and gossypol. The conclusion was that “the ability of FA to considerably prolong survival in nematodes could be related to its property as an inhibitor of Aβ oligomerization” [67].

In order to elucidate the molecular basis of FA destabilizing action on Aβ_(1–40)_ preformed fibrils, the combination of experimental and computational techniques was employed. The experimental part of this study involved: (i) time resolved fluorescence measurements, which can provide information on inter- and intra-molecular interactions; (ii) Fourier transform infrared (FTIR) spectroscopy, which allowed us to follow the conformational changes of Aβ fibrils induced by the incubation with FA; FTIR coupled to hydrogen–deuterium exchange was also applied to follow the effects due to FA on fibril packing and stability; (iii) size exclusion chromatography (SEC), which is able to monitor the formation of soluble species resulting from fibril FA-induced disassemblage; and (iv) confocal microscopy, which gives insight on the morphology of Aβ large aggregates. The experimental measurements were complemented with all atom molecular dynamics (MD) simulations, which are a powerful tool to investigate the conformational characteristics of fibrillar assembly, their constituting oligopeptides, and their interactions with small molecules such as FA. On the basis of these joint experimental and computational studies, it was hypothesized that FA has a dual disrupting action on preformed Aβ fibrils, which is the coating and the partial insertion of FA between the oligopeptides. By means of hydrogen bonding and hydrophobic interactions with the aggregates, FA molecules can bind to and cover Aβ fibrillar structures and they are able to insert their carboxyl and hydroxyl moieties between the oligopeptide chains in close proximity to the bend region (Asp23-Lys28), redirecting the organized conformation of the Aβ fibrils towards amorphous oligomers. It seems that FA molecules induce a sort of stabilization and tightening of the fibrillar structure in the short term, but cause its disruption in the long term. Furthermore, it was observed that FA, interacting with Asp23-Lys28 salt bridges, could disrupt the Asp-Lys arrangement, thus causing an increase in the solvation of this region, the unpacking of the chains, and fibril destabilization. It was speculated that these actions could limit or even hinder the association of incoming Aβ peptides and thus inhibit their re-association in the association-dissociation dynamic equilibrium [68].

Almost simultaneously, two different research groups investigated the effects of FA in the early stages of Aβ fibrillogenesis.

Cui *et al.*, using CD spectroscopy, transmission electron microscopy, and Thioflavin T fluorescence assay, found that FA inhibited the Aβ_(1–42)_ monomer-to-oligomer transition by blocking the formation of the β-sheets but accelerated the Aβ_(1–42)_ oligomer-to-fibril transition. The docking analysis between FA and the amyloid peptide further showed that FA interacts with Aβ_(1–42)_ predominantly by hydrogen bonding with His14 and Glu22, interfering with the formation of β-sheets, thereby inhibiting the formation of aggregates [69].

Bramanti *et al.* [70] studied the *in vitro* effects of FA on Aβ_(1−40)_ early stages of the oligomerization and fibril formation process and on peptide conformational evolution by means of several experimental techniques such as fluorescence and Fourier transform infrared spectroscopy, electrophoresis techniques, chromatographic analysis, and confocal microscopy. It was revealed that FA interacts with Aβ_(1–40)_ in the initial stage of the aggregation process and it interferes with the peptide self-assembly, redirecting it to the formation of non-fibrillar amorphous aggregates. At the molecular level, these complexes possess a β-structure conformation (as witnessed by ThT binding and FTIR), but they are more prone to be destabilized into monomeric species [70].

It is worth noting that these two studies were performed on two different peptides, with Aβ_(1–42)_ being significantly more fibrillogenic than Aβ_(1−40)_, and using a different peptide preparation method. Despite these relevant differences, these results provide a more comprehensive view of the effects of FA on amyloidogenesis related to AD and highlight the intrinsic variability of the assembly process.

## 3. Biological Study on Ferulic Acid

### 3.1. The Oxidative Stress

Several findings in the medical field report the onset of a number of diseases associated with free radical generation. The risk of diseases due to oxidative stress is associated with unhealthy lifestyle, exposure to chemicals, pollution, cigarette smoking, drugs, illness, stress, *etc.*

Oxygen plays a vital role in various biological phenomena, but can also intensify the damage within the cell by oxidative events. Oxygen is the main source of energy and free radicals are formed as a consequence of the adenosine triphosphate (ATP) produced by the mitochondria. Reactive oxygen species (ROS) and reactive nitrogen species (RNS) are the main products obtained by the cellular redox processes. Depending on their delicate balance inside the cells, these reactive species can be beneficial or toxic compounds. At low or moderate levels, reactive species exert beneficial effects on cellular redox signaling and immune function, but at high concentrations, they produce oxidative stress, a harmful process that can damage cell function and structures. Antioxidants are the substance, that can scavenge free radicals and help reduce the incidence of oxidative stress−provoked damage, being able to maintain cellular redox balance [71,72]. Traditional herbal medicines and dietary foods were the main sources of antioxidants for ancient peoples and protected them from the damage caused by free radicals. The new medicine tendencies suggest that the exogenous consumption of antioxidants from plant, animal, and mineral sources can produce beneficial effects on human health and it is effective to reduce the incidence of free radical−induced diseases, including neurodegenerative disorders. For this purpose, an increasing interest in the therapeutic use of antioxidants in the treatment of diseases associated with oxidative stress has been developed [73,74,75,76]. It is, indeed, believed that an antioxidant diet associated with natural antioxidant supplements could protect health from oxidative stress and, consequently, from diseases.

Antioxidants, usable in the treatment of disease, can have natural or synthetic origins. Plants produce an extraordinary number of antioxidant compounds such as carotenoids, flavonoids, cinnamic acids, benzoic acids, folic acid, ascorbic acid, tocopherols, and tocotrienols to prevent oxidation of the susceptible substrate. These plant-based antioxidants are believed to have a better biological effect than the synthetic ones because they contain natural phyto-constituents and, hence, they are believed to have better compatibility with human body.

Chemically synthesized antioxidants are not in nature and are often added to food as preservatives to prevent lipid peroxidation. These antioxidants mainly act via two different ways: as free radical terminators, oxygen scavengers, and chelating agents, or by breaking down the hydroperoxides formed during lipid oxidation into stable end products.

Among the wide classes of compounds, naturally occurring phenols have earned attention and among these, FA in particular.

FA plays a cytoprotective role through the up-regulation of enzymes such as heme oxygenase-1, heat shock protein 70, extracellular signal-regulated kinase (ERK) 1/2, and serine/threonine kinase (Akt). Furthermore, FA inhibits the expression and/or activity of cytotoxic enzymes, including inducible nitric oxide synthase, caspases, and cyclooxygenase-2. All this evidence has encouraged scientists to propose FA for the treatment of several age-related diseases, such as neurodegenerative disorders, cardiovascular diseases, diabetes, and cancer [77].

### 3.2. Ferulic Acid as a Potential Therapeutic Agent for AD

It is well-accepted that oxidative stress contributes to AD neuropathology [78]. Oxidative stress markers have been found increased in neurons surrounding β-amyloid deposits in transgenic mouse models of the disease [79], and experimental induction of oxidative stress leads to Aβ accumulation in primary neurons [80]. ROS are able to oxidize proteins, lipids, and DNA, and increased levels of specific oxidative markers and redox metals have been found in the AD brain [28]. Senile plaque formation in specific regions of the brain induces neuroinflammation and free radical production that contribute to the destruction of brain areas such as the amygdala, hippocampus, and cortex [81].

FA has pleiotropic biological activities, including anti-inflammatory and antioxidant properties, suggesting that long-term administration could delay the progression of AD. It has been, indeed, reported that the long-term administration of FA to mice protected against learning and memory deficits induced by centrally administered β-amyloid [64].

Experiments in which the pre-treatment of FA was followed by the administration of Aβ significantly reversed the neuroinflammation in the mouse hippocampus and ameliorated memory loss, as well evidenced by treatment with Aβ alone [64]. FA meaningfully attenuated peroxyl radical-induced cell death in hippocampal neuronal cells and reduced, in a dose-response manner, both protein oxidation and lipid peroxidation caused by hydroxyl radicals, which are all consequences of oxidative stress [28]. Moreover, FA protects against free radical-mediated changes in conformation of synaptosomal membrane proteins and its effect resulted in being more effective than those achieved by vanillic, coumaric, and cinnamic acid treatments, which are all compounds with similar chemical structures [28].

Cho *et al.* [82] have demonstrated that long-term administration of FA in mice prevented the Aβ_(1–42)_-induced activation of astrocyte cells, which release free radicals and pro-inflammatory cytokines after Aβ activation, resulting in the inflammatory profile of AD, Furthermore, Yan *et al.* [83] have studied the effect of FA administration in female APP/PS1 (APPswe/PS1dE9) transgenic mice, which over express the Swedish mutation of APP together with presenilin 1 (PS1) deleted in exon 9. They found that six months of FA treatment, at the concentration of 5.3 mg/kg/day, significantly reduces the cortical levels of Aβ_(1–40)_ while Aβ_(1–42_) deposition seems slightly affected by FA. The hippocampal deposition of the two peptides did not reach a statistical difference if compared with the controls. The same dosage of FA is able to significantly reduce the interleukin-1β (IL-1β) cortical levels [83].

In the context of neurological disease, intravenous administration of FA has been shown to protect against neuronal cell death induced by cerebral ischemia [84,85,86]. Interestingly, FA is also known to stimulate neural progenitor cell proliferation *in vitro* and *in vivo*, which has been demonstrated to ameliorate stress-induced depression-like behavior in mice [87]. On the basis of these results, it was tested whether FA can attenuate AD-like pathology in a transgenic mouse model of cerebral amyloidosis [88]. After a six-month treatment, FA reversed behavioral impairment, including hyperactivity, object recognition, spatial working, and reference memory. Moreover, it reduced amyloidogenic APP metabolism by modulation of β-secretase, attenuated neuroinflammation, and stabilized oxidative stress.

### 3.3. FA Influences Cell Signaling and Apoptosis

A tight consequence of oxidative stress is apoptotic cascade activation. Apoptosis is controlled by different proteins of the kinase family, such as the mammalian mitogen–activated protein kinases (MAPKs) [89]. MAPKs are the family of kinases that transduce signals from the cell membrane to the nucleus in response to a wide range of stimuli, including stress. MAPKs are serine/threonine kinases that, upon stimulation, phosphorylate their specific substrates at serine and/or threonine residues. Such phosphorylation events can either positively or negatively regulate the substrate and the entire signaling cascade activity. Thus, the MAPK signaling pathways modulate gene expression, mitosis, proliferation, motility, metabolism, and the programmed cell death. This kind of kinase consists of different family members: the extracellular signal-regulated kinases (ERK1/2), and the stress-activated protein kinases, including Jun N-terminal kinase (JNK) and p38-mitogen-activated protein kinase (p38-MAPK) [90,91]. However, all these kinases are involved in regulation of apoptosis.

In addition, when cells are exposed to growth factors such as insulin and certain cytokines, other kinases, including serine/threonine kinase Akt, activated via the phosphoinositide 3-kinase (PI3K)-dependent signaling pathway, play an anti-apoptotic role [92].

The intracerebroventricular injection of Aβ in rats produces a significant increase in activated p38 MAPK and IL-1β expression in the hippocampal cornus ammonis1 (CA1) brain region. This effect was counteracted by sodium ferulate (SF), a FA-like molecule [93]. SF is also able to revert the Aβ-induced inactivation of ERK1/2 and to increase the activation of Akt [93]. Moreover, SF attenuated the Aβ-induced caspase activation. The injection of Aβ in rats activated the caspase-9, caspase-7, and casapse-3 cascade that was inhibited by the pre-treatment with SF, indicating that SF contributed to prevent neurotoxicity [94].

FA has been found to enhance the cell stress response by regulating several key cytoprotective enzymes, e.g., heme oxygenase-1, heat shock protein 70, the heme oxygenase/biliverdin reductase (HO/BVR) system, superoxide dismutase (SOD), catalase (CAT) ERK 1/2, and Akt [95]. The main mechanism of action of these enzymes is, indeed, to counteract the free radical-induced damage. In contrast, FA was shown to inhibit the expression and/or activity of cytotoxic enzymes, including inducible nitric oxide synthase, caspases, and cyclooxygenase-2 [95].

### 3.4. Nanotechnology for FA Delivery

A central problem in the treatment of brain disorders is to deliver a suitable drug amount into the brain, which is due to the obstacle of the blood brain barrier (BBB) against the entry of a variety of molecules into the CNS tissue. A good strategy to increase the bioavailability and the cytoprotective effect of compounds such as FA is the formulation of new nanoparticles. Nanotechnology has shown promising applications in targeted drug delivery for AD, and several nanocarrier systems have been studied in recent years to increase the bioavailability and efficacy of different AD therapeutic agents [91]. The use of nanoparticles has many advantages, including the possibility of controlled drug release and drug targeting, increased drug stability, high drug loading capacity, the feasibility of incorporating lipophilic and hydrophilic drugs, the biocompatibility of the carrier, and no problems with respect to large-scale production and sterilization [96]. Due to their small size, these systems may be injected intravenously, avoiding the uptake of macrophages of the mononuclear phagocyte system (MPS). Furthermore, their lipophilic features could lead them to the CNS, crossing the BBB by means of the endocytotic mechanism of the endothelial cells lining the brain’s blood capillaries. Among the different formulations, nanoparticles made from solid lipids, called solid lipid nanoparticles (SLNs), have been employed to improve the delivery of antioxidant agents [58,59]. SLNs are considered one of the safer nanoparticle systems suggested for drug delivery to the brain. In the study of Picone *et al.* [58], SLNs were formulated and successfully employed as a nanocarrier for FA. SLNs were prepared via a warm oil-in-water microemulsion by using Compritol 888 ATO under mechanical stirring, and FA-loaded SLNs were obtained by dissolving FA in the melted lipid matrix. The resultant nanocomplex possesses a small colloidal surface with a highly negative surface charge when dispersed in water. The effect of FA, free or loaded, on SLNs was tested on neuroblastoma cells, previously treated with Aβ_(1–42)_ oligomers. FA reduced ROS production, normalized mitochondrial membrane potential, and decreased cytochrome C release, leading to a recovery of the compromised cell viability [58]. Enhancing the drug stability and uptake, the SLN nanocarrier improved the antioxidant effects of FA with respect to free FA [58,59].

Moreover, FA was also used to produce a particular nanoparticle. By the chemical conjugation of FA to water-soluble glycol chitosan (GC), hydrophobically self-assembled nanoparticles, composed of a hydrophobic FA core and a hydrophilic GC shell, were formulated [97]. Using a spinal cord contusion injury model, significant recovery in locomotor function was observed in rats in which nanoparticles were intravenously administered. Histological analysis revealed that FA-GC treatment significantly preserved axons and myelin and also reduced cavity volume, astrogliosis, oxidative stress, and inflammatory response at the lesion site, suggesting an improvement of the neuroprotective effect of the utilized molecules.

Stearic acid- and stearyl ferulate-based solid lipid nanoparticles containing *trans*-ferulic acid were prepared and characterized for loading. Efficiency, size, and shape and their antioxidant activity were evaluated. It was suggested that the obtained formulations facilitate the uptake of FA by the cells on the basis of their lipophilic structure, thus increasing FA bioavailability [98]. Furthermore, stearyl ferulate-based nanoparticles could prevent the degradation of FA entrapped in their structure, making FA almost entirely available to explicate its antioxidant power once released. However, these new formulations significantly enhanced the antioxidant effects of FA against both ROS and RNS in preclinical models [58,98] and are considered a useful platform for the possible translation of this strategy to clinical evaluation. In this regard, it is very important to underline that favorable preclinical results should overcome the clinical tests to receive a positive therapeutic evaluation and be considered risk-free [99].

## 4. Conclusions

The aim of the present review is to illustrate the potential of FA as a therapeutic agent against neurodegeneration, in particular against damage caused by AD. FA has given positive results in the inhibition of neurotoxic Aβ-aggregation *in vitro* and *in vivo* in animal models. Furthermore, FA is able to interfere with the biological pathways involved in apoptotic programmed cell death induced by oxidative stress and inflammation due to Aβ aggregation. The obtained results encourage the use of FA as a drug against neurodegenerative diseases, but many questions are still to be answered before considering FA a safe therapeutic agent to be administered to patients. In the case of humans, which dose should be used in order to obtain effects comparable with those observed in the *in vitro* experiments? Does the administration of FA cause damage in the kidney or liver due to its possible accumulation? At the present time, the therapeutic use of FA still remains to be explored.
